# Lambda Light Chain Multiple Myeloma in a 47-Year-Old Female Patient on Long-Term Clozapine Therapy

**DOI:** 10.7759/cureus.92079

**Published:** 2025-09-11

**Authors:** Riya Goel, Justin Liu, Katherine Jordak, Manpreet Malik

**Affiliations:** 1 Internal Medicine, Emory University School of Medicine, Atlanta, USA; 2 Hospital Medicine, Emory University School of Medicine, Atlanta, USA

**Keywords:** chronic clozapine use, clozapine, hematological malignancy, lambda light chain, multiple myeloma, schizophrenia

## Abstract

Clozapine is an atypical antipsychotic used for treatment-resistant schizophrenia and schizoaffective disorder. While its hematologic toxicities, such as neutropenia and agranulocytosis, are well-documented, emerging studies suggest a rare but concerning association between long-term clozapine use and hematologic malignancies. We present a case of multiple myeloma (MM) in a 47-year-old woman with a 10-year history of clozapine use for schizoaffective disorder. The patient was admitted with orthostasis, fatigue, and acute kidney injury following a gastrointestinal illness. Initial workup revealed severe anemia, a pelvic mass later attributed to uterine fibroids, and extensive lytic bone lesions on imaging. Hematologic evaluation demonstrated a markedly elevated lambda free light chain level, suppressed kappa/lambda ratio, and rouleaux formation on peripheral smear. Bone marrow biopsy confirmed 90% lambda light chain-restricted plasma cells, consistent with MM. The patient responded to cyclophosphamide-bortezomib-dexamethasone (CyBorD) chemotherapy and was discharged to continue outpatient treatment. This case underscores the importance of maintaining clinical vigilance for hematologic malignancies in patients on long-term clozapine therapy, particularly when presenting with unexplained renal dysfunction, anemia, or lytic bone lesions. Although leukemias and lymphomas are reported more than MM, this case adds to the limited literature describing MM in the context of chronic clozapine exposure and highlights the need for further pharmacovigilance and research into long-term hematologic risks of atypical antipsychotics.

## Introduction

Clozapine is a well-known antipsychotic medication used for conditions such as treatment-resistant schizophrenia and schizoaffective disorder. However, its use is limited by serious adverse effects such as agranulocytosis, myocarditis, and metabolic syndrome [1]. Although clozapine remains the most effective antipsychotic for treatment-resistant schizophrenia, emerging evidence suggests a rare but notable link between long-term use and hematologic malignancies [2,3]. A study conducted in Finland among patients with schizophrenia found that cumulative clozapine exposure of ≥5000 defined daily doses was associated with a significantly increased risk of hematological malignancies (odds ratio or OR 3.35; 95% CI, 2.22-5.05; p<0.0001) [2]. Another large population-based study among veterans with schizophrenia found a dose-dependent association between cumulative clozapine exposure and increased risk of hematologic cancers, including leukemias, lymphomas, and myelomas [3].

Despite significant literature describing clozapine-related neutropenia and agranulocytosis [4,5], only isolated case reports, particularly of non-secretory multiple myeloma (MM) in this context, have been documented [6]. MM typically affects older adults, the average age of diagnosis in the US being 69 years [7]. It also impacts males more than females [8]. Therefore, the discovery of this plasma cell malignancy in the setting of long-term clozapine use in younger patients is noteworthy and raises important questions about causality and surveillance. We report a case of MM diagnosed in a 47-year-old female patient after a decade of clozapine therapy, exploring the correlation and advocating for heightened clinical vigilance. 

## Case presentation

A 47-year-old African-American woman with a past medical history of schizoaffective disorder-bipolar type (diagnosed in 2016), type 2 diabetes mellitus (diagnosed in 2016), well-controlled on oral medications (A1C 5.9%), hypertension (diagnosed in 2021), and chronic iron-deficiency anemia (diagnosed in 2016, on oral iron supplementation) presented to the Emergency Department with orthostasis and dizziness for one week following resolution of a gastrointestinal illness characterized by nausea, vomiting, and diarrhea. She reported 10-15 lbs of unintentional weight loss over two weeks. She also endorsed a history of heavy menstrual bleeding during her last cycle. Otherwise, she had regular, moderately heavy periods, typically lasting seven days per cycle. Her home medications included clozapine 300 mg nightly (on therapy for nine years), fluoxetine 20 mg daily (since 2016), carvedilol 25 mg twice daily, losartan 25 mg daily, atorvastatin 40 mg daily, and oral iron supplements 325 mg every other day. She was on dapagliflozin and semaglutide for diabetes. She had no family history of hematologic malignancy. She was single, with one child, and employed in a kitchen. She denied tobacco, alcohol, or illicit drug use. 

**Figure 1:**
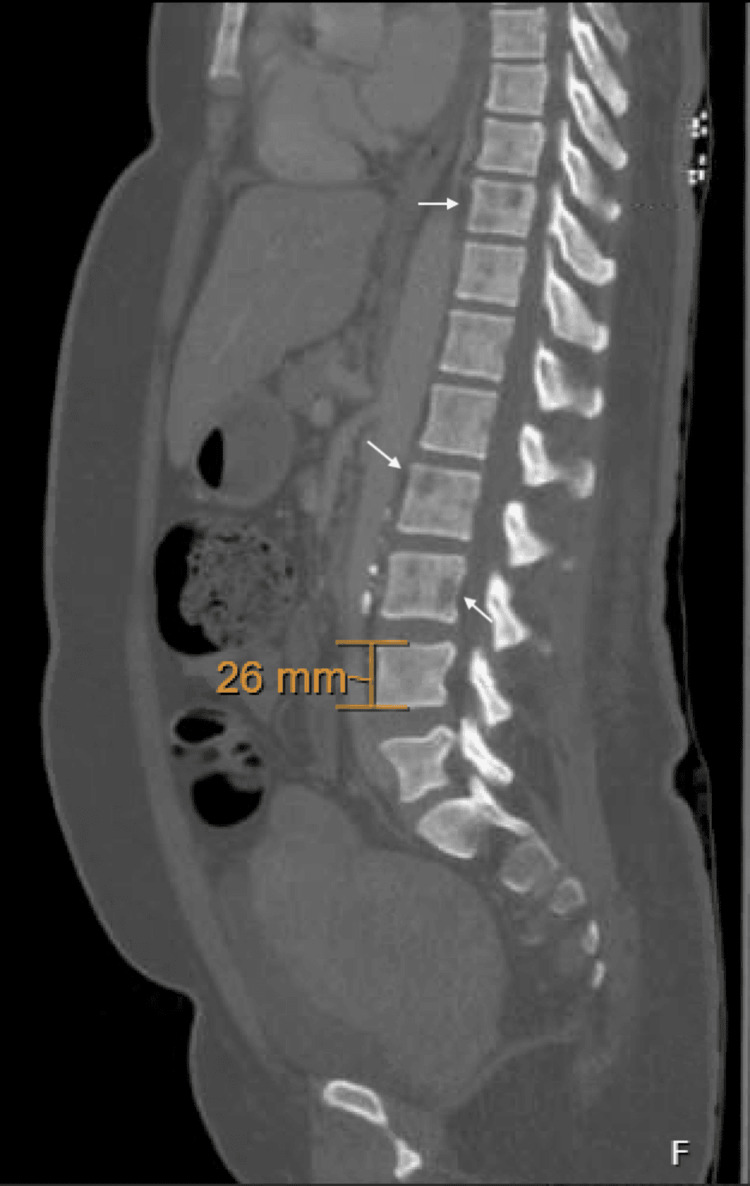
CT scan of the abdomen and pelvis (sagittal view) demonstrating innumerable lytic lesions on multiple levels of vertebral bodies (white arrows)

**Figure 2:**
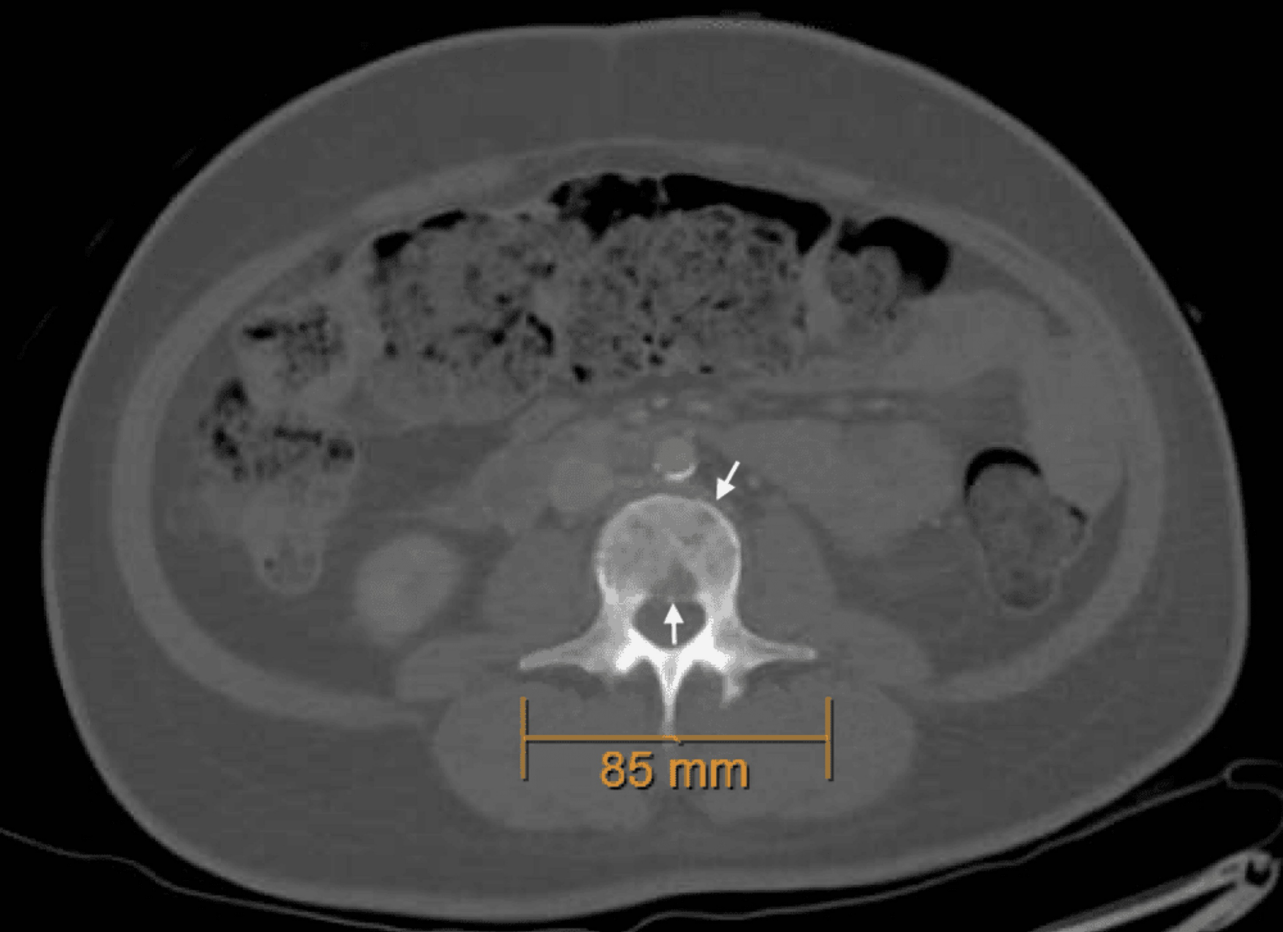
CT scan of the abdomen and pelvis (axial view) demonstrating innumerable lytic lesions on the vertebral body (white arrows)

On admission, her vitals were temperature 36.9 °C (98.4 °F), heart rate 99 beats per minute, respirations 16 breaths per minute, blood pressure 126/71 mmHg, and SpO2 99%. Physical exam revealed pallor and orthostatic changes in blood pressure, without lymphadenopathy, hepatosplenomegaly, or bone tenderness. Initial labs demonstrated acute kidney injury with creatinine of 3.4 mg/dL (baseline 1.0 mg/dL one month prior) and blood urea nitrogen (BUN) of 25 mg/dL. Intravenous hydration with normal saline at 100 ml/hour produced minimal improvement (creatinine 3.1 mg/dL on the following day). Hemoglobin was 6.5 g/dL (baseline 8.3 mg/dL three months prior), and she received one unit of packed red blood cells, after which hemoglobin improved to 8.2 mg/dL. A pelvic ultrasound revealed a complex right adnexal mass and multiple uterine fibroids, the largest measuring 11 × 9.5 × 6.8 cm. Her tumor markers were negative. CT imaging of the abdomen/pelvis showed innumerable lytic bone lesions (Figures 1, 2).

An MRI confirmed diffuse marrow signal abnormality, and the adnexal mass was determined to be from fibroids. 

**Figure 3:**
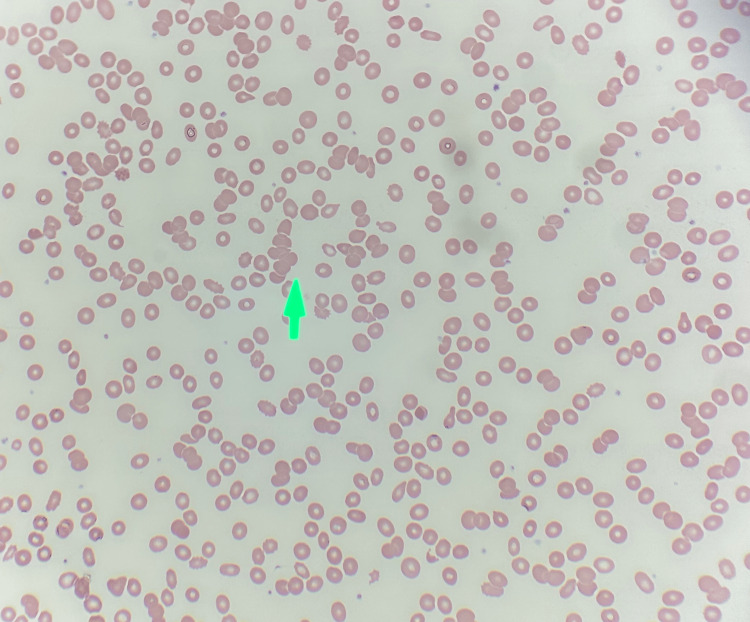
Rouleaux formation on the blood smear (green arrow)

Further workup revealed a protein gap of 5.5 g/dL (defined as the difference between serum total protein and albumin; elevated gaps >4 g/dL suggest monoclonal gammopathy) [9], serum calcium 8.4 mg/dL, and peripheral smear with rouleaux formation (red blood cell stacking due to elevated positively charged plasma proteins) [10] (Figure 3).

Serum free light chain analysis showed lambda 2,536 mg/L and kappa 22.9 mg/L with a suppressed kappa/lambda ratio of 0.01. Urine studies demonstrated free lambda light chains with 24-hour proteinuria of 1,696 mg. Immunoglobulin quantification revealed depressed IgM (<20 mg/dL) and IgA (18 mg/dL) with elevated IgG (3,475 mg/dL) (Table 1).

**Table 1:** Laboratory values during admission

Test	Patient value	Reference range
Protein gap	5.5 g/dL	<4.0 g/dL
Serum calcium	8.4 mg/dL	8.6-10.3 mg/dL
Creatinine (on admission)	3.4 mg/dL	0.6-1.3 mg/dL
Creatinine (next day)	3.1 mg/dL	0.6-1.3 mg/dL
Blood Urea Nitrogen (BUN)	25 mg/dL	7-20 mg/dL
Hemoglobin	6.5 g/dL	12.0-16.0 g/dL (female)
Kappa free light chains (initial)	22.9 mg/L	3.3-19.4 mg/L
Lambda free light chains (initial)	2,536 mg/L	5.7-26.3 mg/L
Kappa/Lambda ratio (initial)	0.01	0.26-1.65
24-hour urine protein	1,696 mg/24 hr	<150 mg/24 hr
Immunoglobulin G	3,475 mg/dL	700-1,600 mg/dL
Immunoglobulin A	18 mg/dL	70–400 mg/dL
Immunoglobulin M	<20 mg/dL	40-230 mg/dL
Carcinoembryonic Antigen	<5 ng/mL	<5 ng/mL (non-smoker)
Inhibin A/B, Carbohydrate Antigen 19-9, Cancer Antigen 125	Negative	Within respective normal limits

Bone marrow biopsy showed hypercellular marrow (90%) with plasma cell infiltration comprising 90% of total cells, lambda light chain restriction on flow cytometry, and negative Congo red staining for amyloid. 

The patient was diagnosed with MM, Revised International Staging System (RISS) stage III [11], and initiated first-line chemotherapy with cyclophosphamide-bortezomib-dexamethasone (CyBorD) on a 21-day cycle (IV cyclophosphamide 900 mg/m² on day one, SQ bortezomib 1.3 mg/m² on days one, four, eight, and 11, and PO dexamethasone 40 mg on days one, two, four, eight, and 11). Over the first week of admission, lambda levels decreased from 2,536 to 1,357 mg/L, and she experienced symptomatic improvement in fatigue and orthostasis. She was discharged after the initial inpatient treatment with prophylactic acyclovir 400 mg BID and allopurinol 100 mg daily, PO dexamethasone 40 mg with chemotherapy treatments, and her home clozapine 300 mg nightly. 

She continued outpatient CyBorD therapy for approximately one month before transitioning to induction with Daratumumab, lenalidomide, bortezomib, and dexamethasone (Dara-RVD) on a 21-day cycle, consisting of subcutaneous daratumumab-hyaluronidase 1,800 mg on days one, eight, and 15; SQ bortezomib 1.3 mg/m² on days one, four, eight, and 11; PO lenalidomide 10 mg on days one to 14; and PO dexamethasone 40 mg weekly, with aspirin for thromboprophylaxis, as well as acyclovir and trimethoprim-sulfamethoxazole for herpes simplex and pneumocystis jirovecii infection prophylaxis, respectively. 

She has continued close follow up with hematology and also has regular visits with her psychiatrist. At her most recent hematology follow-up, approximately two months after discharge, she remained clinically stable on Dara-RVD (bortezomib, dexamethasone, and lenalidomide 10 mg nightly for 14 days) and ongoing revlimid 10 mg daily. Her other medications included home psychiatric and cardiovascular therapy of clozapine 200 mg nightly (decreased from admission due to concern that recent gastrointestinal illness and poor kidney function could lead to supratherapeutic clozapine levels), fluoxetine 40 mg daily, losartan 25 mg daily, carvedilol 6.25 mg twice daily, and atorvastatin 40 mg daily, as well as diabetes treatment with dapagliflozin 10 mg daily. Supportive medications included acyclovir 400 mg daily, allopurinol 100 mg daily, and PRN oxycodone 5 mg every six hours for pain and prochlorperazine 10 mg every six hours for nausea. This combination regimen will be maintained for approximately four months, after which she is scheduled to transition to bone marrow transplantation with an anticipated two-week inpatient stay.

## Discussion

This case highlights lambda light chain multiple myeloma (LCMM) in a 47-year-old woman on long-term clozapine therapy. MM is a malignancy of plasma cells, characterized by the clonal proliferation of cells producing either intact immunoglobulins or free light chains, typically diagnosed when ≥10% of bone marrow plasma cells are present along with end-organ damage, such as hypercalcemia, renal impairment, anemia, or bone lesions [12]. LCMM is a less common subtype, accounting for roughly 15% of MM cases, in which malignant plasma cells secrete only light chains without immunoglobulin production. This form often presents with renal dysfunction, anemia, and lytic bone disease and generally has a more aggressive clinical course [12]. Our patient fulfilled the International Myeloma Working Group criteria with >90% lambda-restricted plasma cells, positive serum free light chain ratio, anemia, renal impairment, and extensive lytic bone lesions [13]. 

Clozapine is well known for its hematologic side effects, particularly agranulocytosis (incidence 0.9%) and neutropenia (0.8-2%), and, rarely, pancytopenia [1]. Experimental studies suggest a potential mechanism for marrow dysregulation, as clozapine metabolites can inhibit the hematopoietic precursor cell growth in a dose-dependent manner, affecting multiple lineages [14]. While most reports focus on transient cytopenias, prolonged or cumulative marrow stress could theoretically contribute to clonal plasma cell proliferation, as seen in LCMM [12]. More recently, clozapine has been linked to hematologic malignancies: a Finnish nationwide study reported increased risk of leukemias, lymphomas, and myelomas with cumulative clozapine exposure ≥5,000 defined daily doses [2], and a U.S. veteran cohort study demonstrated a dose-dependent increase in hematologic cancer risk, with cumulative exposures of 3,000-4,999 defined daily doses associated with an OR of 1.78 (95% CI, 1.13-2.79) and exposures ≥5,000 defined daily doses with an OR of 1.81 (95% CI, 1.24-2.64) [3]. Our patient had a cumulative exposure of approximately 3,285 defined daily doses. Case reports have described clozapine-associated leukemias and lymphomas, with isolated reports of non-secretory myeloma [6]. Our patient adds to this literature as a case of LCMM following a decade of clozapine therapy. Her relatively young age, female sex, and absence of hypercalcemia diverge from typical myeloma demographics, support the possibility that chronic clozapine-induced marrow dysregulation contributed to her disease. 

Compared to other atypical antipsychotics, clozapine carries the highest risk for hematologic dyscrasias. Clozapine use was associated with a significantly increased incidence of hematologic malignancies compared to olanzapine, with a weighted incidence rate ratio (IRR) of 2.22 (95% CI: 1.52-3.34; p<0.001) [15]. Notably, olanzapine and risperidone have no consistent association with plasma cell malignancies [16,17]. In addition, pharmacovigilance disproportionality analyses have identified elevated reporting OR for lymphoid malignancies with clozapine (e.g., reporting OR=3.76 for Hodgkin lymphoma and reporting OR=3.62 for non-Hodgkin lymphoma), whereas comparable signals have not been observed for several other atypical agents [18]. Taken together with the population-based cohort and registry studies showing dose-dependent associations, these data strengthen a signal that clozapine, more so than other atypicals, may be linked to hematologic malignancies. However, spontaneous-reporting analyses are subject to reporting bias and cannot establish incidence or causation, so these signals require confirmation in controlled epidemiologic studies [2,3,13-16]. 

The American Psychiatric Association requires regular hematological monitoring for agranulocytosis and neutropenia during clozapine therapy, but does not recommend routine screening for hematological malignancies, including MM or its subtypes [19]. While the elevated risk is recognized, given absolute risk is not considered high, there are currently no routine surveillance guidelines [2,18]. This report contributes to the growing awareness of potential long-term hematologic consequences of clozapine [2,3,6,16], and advocates pharmacovigilance in atypical antipsychotic therapy [20]. 

The temporal association between chronic clozapine use and onset of MM in this relatively young female patient raises clinical concern. Her atypical presentation, without classic myeloma bone pain or pronounced hypercalcemia, underscores the heterogeneity of MM presentation, especially in patients with overlapping comorbidities [21]. This case raises important questions regarding surveillance in long-term clozapine users. Systematic studies are needed to clarify whether duration or cumulative dose of clozapine independently predicts plasma cell malignancies, whether women are at particular risk, and whether hematologic screening beyond standard complete blood count (CBC) monitoring is warranted. Prospective pharmacovigilance studies and pooled registry analyses may help define at-risk populations and guide early detection strategies. 

## Conclusions

This case contributes novel insights into the rare but emerging association between chronic clozapine use and plasma cell dyscrasias, specifically LCMM. Unlike previous reports that predominantly describe leukemias and lymphomas, this case adds to the limited literature linking atypical antipsychotics with plasma cell malignancies. The patient’s younger age, female sex, and lack of hallmark features such as hypercalcemia highlight the atypical clinical presentation that may delay diagnosis. It calls for heightened pharmacovigilance and consideration of MM in differential diagnoses when patients on long-term clozapine present with renal dysfunction, anemia, or bone pain, even in the absence of classic findings. Further research is needed to elucidate the mechanisms of clozapine-associated marrow dysregulation and to evaluate whether targeted surveillance guidelines are warranted for high-risk patients.
